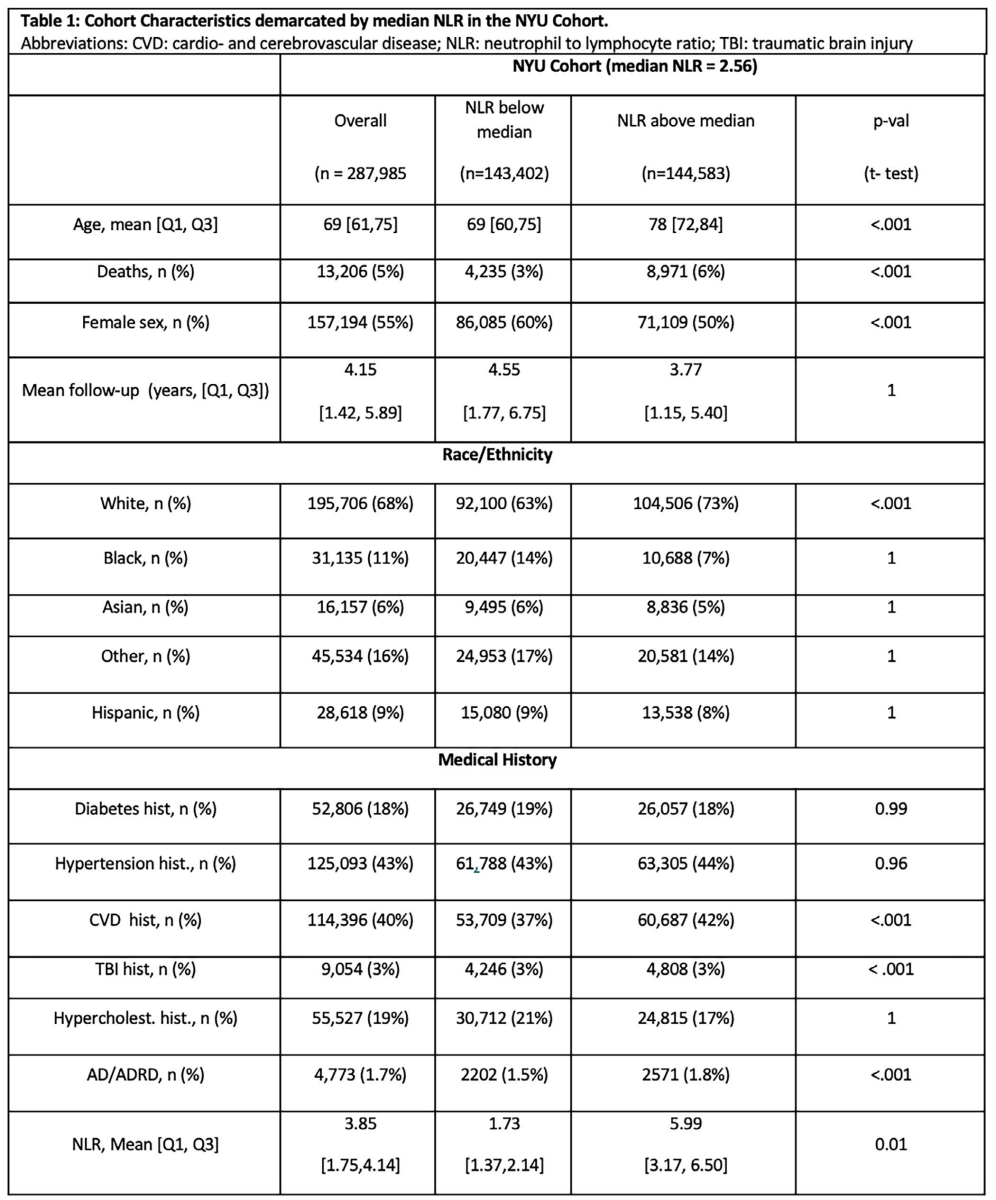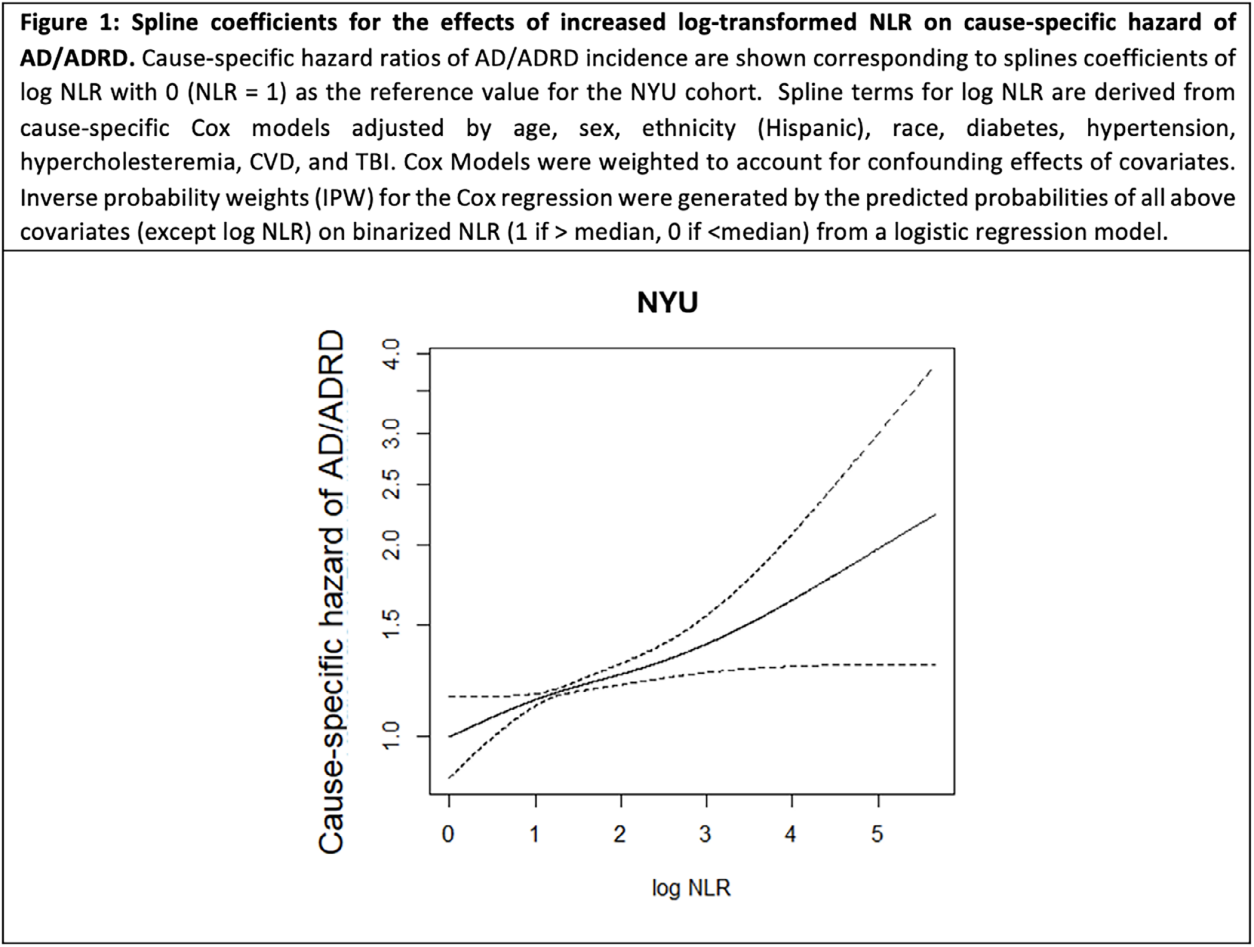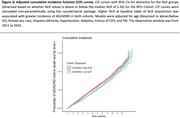# Neutrophil inflammation metrics are associated with the risk of future dementia in large‐scale electronic health record data from hospital systems

**DOI:** 10.1002/alz70855_107044

**Published:** 2025-12-24

**Authors:** Mark He, Sean R Jacobson, Rebecca A Betensky, Ricardo S. Osorio, Tovia Jacobs, Ula Hwang, Omonigho M Bubu, Thomas Wisniewski, Jaime Ramos Cejudo

**Affiliations:** ^1^ New York University (NYU) Grossman School of Medicine, New York City, NY, USA; ^2^ New York University (NYU) Grossman School of Medicine, New York, NY, USA; ^3^ NYU College of Global Public Health, New York, NY, USA; ^4^ NYU Grossman School of Medicine, New York, NY, USA; ^5^ NYU Langone Health, New York, NY, USA; ^6^ New York University Grossman School of Medicine, New York, NY, USA

## Abstract

**Background:**

Neutrophils play a role in Alzheimer's disease (AD) pathology and AD‐related dementias (AD/ADRD), and prior research has shown that the neutrophil to lymphocyte ratio (NLR), a marker of neutrophil‐mediated inflammation, is associated with the risk of future dementia. To date, studies looking at this relationship have used small cohorts. We address whether this is generalizable to larger populations using electronic health records (EHR) data from all six sites of NYU Langone Hospitals

**Method:**

Our study window ranged from 2011‐2023. NLR values were obtained from laboratory test results. For each patient, the first NLR obtained was used; the associated date was taken to be the time origin. The outcome was AD/ADRD incidence, defined using ICD‐codes over the same study window at least 6 months post‐baseline. Cause‐specific Cox regression was used to determine the independent association of log‐transformed NLR values with the risk of future AD/ADRD adjusting for demographic and clinical confounders and with death as a censoring event. To account for confounding effects of covariates, the Cox model was weighted by the predicted probabilities of all adjusting covariates on binarized NLR (“high” if >median, “low” if <median) from a logistic model. We used cumulative incidence function (CIF) curves to assess the risk over time stratified by binarized NLR accounting for the competing risk of death. Sex‐ and race‐ subgroup analysis was also conducted.

**Result:**

The study sample included 287,985 patients at NYU (4,773 AD/ADRD cases, mean age [Q1, Q3] = 69 [61, 75], 55% female, 11% black/AA). We found a positive and independent association of log NLR with cause‐specific hazard of AD/ADRD (HR = 1.160 with 95% CI [1.100, 1.223]. Spline terms for log NLR indicated significant increasing relationships with cause‐specific hazard of AD/ADRD. Subgroup analyses showed consistently significant associations, with higher hazard among Hispanic patients. CIF curves showed meaningful separation for high vs low‐NLR groups.

**Conclusion:**

Our findings suggest that individuals with higher NLR are at greater risk of future AD/ADRD regardless of the study cohort or demographics, reinforcing the importance of peripheral inflammation and the need to clarify the mechanisms by which neutrophil biology influences AD pathology